# Supplementary material for: SARS-CoV-2 Omicron (B.1.1.529) Infection of Wild White-Tailed Deer in New York City
Source: Viruses. 2022 Dec 12;14(12):2770. doi: 10.3390/v14122770 (PMC9785669; doi:10.3390/v14122770)
Supplement: Supplementary file 1 [file viruses-14-02770-s001.zip › viruses-1986517-supplementary.pdf]

Supplementary Table S1. Metadata associated with the deer samples collected from Staten Island, NY.

ID	Capture Date	Age (years)	Sex	RT-PCR result	RT-PCR Ct	sVNT result	sVNT (inhibition %)	pVNT (NT_{50})					
								Wild type B.1	Alpha	Beta	Gamma	Delta	Omicron
**80**	12/12/2021	0.5	M	ND	ND	NEG	3.73						
**90**	1/11/2022	0.5	F	NEG	UN	NEG	10.58						
**97**	1/19/2022	0.5	F	ND	ND	NEG	7.44						
**100**	1/9/2022	0.5	F	NEG	UN	NEG	8.89						
**101**	1/20/2022	3.5	F	NEG	UN	NEG	7.71						
**102**	1/6/2022	0.5	F	POS	30.64	ND	ND						
**103**	1/6/2022	0.5	F	POS	32.14	NEG	8.85						
**104**	1/6/2022	0.5	F	POS	35.57	NEG	7.81						
**106**	1/27/2022	2.5	F	NEG	UN	NEG	22.69						
**120**	12/18/2021	0.5	F	ND	ND	NEG	10.99						
**121**	1/9/2022	0.5	F	ND	ND	NEG	11.31						
**123**	1/5/2022	0.5	F	NEG	UN	NEG	2.56						
**1584**	12/12/2021	3.5	M	ND	ND	POS	74.72	281.2	197.9	34.19	43.53	76.23	ND
**2002**	12/15/2021	1.5	M	ND	ND	NEG	28.82						
**2003**	12/14/2021	2.5	M	ND	ND	NEG	2.5						
**2004**	12/13/2021	1.5	M	ND	ND	POS	93.97	1681	849.6	282.2	545.8	595.8	247.5
**2005**	12/13/2021	0.5	M	ND	ND	NEG	8.4						
**2006**	12/29/2021	0.5	M	ND	ND	NEG	2.5						
**2007**	12/29/2021	0.5	M	ND	ND	NEG	3.08						
**2008**	12/15/2021	1.5	M	ND	ND	NEG	0.36						
**2009**	12/15/2021	1.5	M	ND	ND	NEG	-2.11						
**2010**	12/15/2021	0.5	M	ND	ND	NEG	2.82						
**2011**	12/16/2021	1.5	M	ND	ND	NEG	3.01						
**2012**	12/16/2021	0.5	M	ND	ND	NEG	3.27						
**2013**	12/15/2021	0.5	M	ND	ND	NEG	3.47						
**2014**	12/20/2021	1.5	M	ND	ND	NEG	4.51						

ID	Capture Date	Age (years)	Sex	RT-PCR result	RT-PCR Ct	sVNT result	sVNT (inhibition %)	pVNT (NT_{50})					
								Wild type B.1	Alpha	Beta	Gamma	Delta	Omicron
**2015**	12/17/2021	0.5	M	ND	ND	POS	87.75	340	157.3	64.05	67.17	1028	55.79
**2016**	12/18/2021	0.5	M	ND	ND	NEG	-6.84						
**2018**	12/18/2021	1.5	M	ND	ND	NEG	9.82						
**2019**	12/19/2021	2.5	M	ND	ND	NEG	8.14						
**2020**	12/16/2021	0.5	M	ND	ND	NEG	2.3						
**2021**	12/19/2021	0.5	M	ND	ND	NEG	0.75						
**2022**	12/17/2021	0.5	M	ND	ND	NEG	5.28						
**2023**	12/22/2021	1.5	M	ND	ND	NEG	3.92						
**2025**	12/18/2021	1.5	M	ND	ND	POS	92.35	606.3	337.8	51.24	107.2	1228	25.91
**2026**	12/18/2021	0.5	M	ND	ND	NEG	3.73						
**2028**	12/20/2021	0.5	M	ND	ND	NEG	6.77						
**2029**	12/20/2021	0.5	M	ND	ND	NEG	1.52						
**2031**	12/22/2021	0.5	M	ND	ND	NEG	2.5						
**2032**	12/21/2021	0.5	M	ND	ND	NEG	2.24						
**2033**	12/31/2021	1.5	M	ND	ND	POS	68.62	490.4	215.5	126.8	149.8	260.7	50.23
**2034**	12/30/2021	0.5	M	ND	ND	NEG	3.21						
**2035**	12/21/2021	1.5	M	ND	ND	POS	33.16	169.5	132.6	123.5	80.91	127	19.18
**2036**	12/28/2021	1.5	M	ND	ND	POS	60	157	153.1	66.79	145.4	127.2	44.03
**2038**	12/22/2021	0.5	M	ND	ND	NEG	15.59						
**2039**	12/29/2021	0.5	M	ND	ND	NEG	19.55						
**2040**	12/30/2021	1.5	M	ND	ND	POS	51.83	144.4	95.13	86.28	124.8	194.5	62.82
**2041**	12/30/2021	1.5	M	ND	ND	NEG	10.08						
**2042**	12/31/2021	0.5	M	ND	ND	NEG	11.31						
**2043**	1/1/2022	0.5	M	ND	ND	NEG	2.5						
**2044**	12/31/2021	1.5	M	ND	ND	NEG	1.07						
**2045**	12/30/2021	1.5	M	ND	ND	POS	46.97	347.7	182.8	175.4	194.4	137.7	30.65
**2047**	1/2/2022	2.5	M	ND	ND	NEG	16.24						
**2048**	1/3/2022	0.5	M	NEG	UN	NEG	10.47						

ID	Capture Date	Age (years)	Sex	RT-PCR result	RT-PCR Ct	sVNT result	sVNT (inhibition %)	pVNT (NT_{50})					
								Wild type B.1	Alpha	Beta	Gamma	Delta	Omicron
**2049**	1/4/2022	1.5	M	NEG	UN	NEG	15.82						
**2050**	12/31/2021	1.5	M	ND	ND	NEG	3.47						
**2051**	1/3/2022	1.5	M	NEG	UN	NEG	11.31						
**2052**	1/1/2022	1.5	M	ND	ND	NEG	1.85						
**2053**	1/4/2022	1.5	M	NEG	UN	NEG	7.23						
**2054**	1/4/2022	0.5	M	NEG	UN	NEG	0.94						
**2055**	1/4/2022	1.5	M	NEG	UN	POS	97.02	2406	1718	1741	1427	1526	273.6
**2056**	1/7/2022	0.5	M	NEG	UN	NEG	3.29						
**2057**	1/2/2022	0.5	M	ND	ND	NEG	6.06						
**2058**	1/2/2022	0.5	M	ND	ND	NEG	8.14						
**2059**	1/7/2022	3.5	M	NEG	UN	NEG	4.64						
**2060**	1/2/2022	1.5	M	ND	ND	NEG	6.04						
**2061**	1/8/2022	0.5	M	NEG	UN	NEG	7.82						
**2062**	1/5/2022	1.5	M	NEG	UN	POS	70.89	337.1	145.5	142.4	187.2	278.8	119.3
**2063**	1/6/2022	1.5	M	NEG	UN	ND	ND						
**2064**	1/5/2022	0.5	M	NEG	UN	POS	69.66	100.3	73.54	32.34	34.38	313.2	28.5
**2065**	1/6/2022	0.5	M	NEG	UN	ND	ND						
**2066**	1/7/2022	1.5	M	NEG	UN	NEG	-0.8						
**2067**	1/7/2022	0.5	M	POS	36.3	NEG	7.36						
**2068**	1/9/2022	0.5	M	NEG	UN	ND	ND						
**2070**	1/6/2022	1.5	M	ND	ND	NEG	8.91						
**2071**	1/8/2022	2.5	M	NEG	UN	POS	96.37	1916	1329	396.8	276.7	1803	77.12
**2072**	1/13/2022	0.5	M	NEG	UN	NEG	14.42						
**2073**	1/10/2022	0.5	M	NEG	UN	NEG	12.8						
**2074**	1/8/2022	0.5	M	NEG	UN	NEG	7.47						
**2075**	1/14/2022	1.5	M	NEG	UN	POS	78.48	360.3	417	106.8	73.32	245.5	44.24
**2076**	1/9/2022	0.5	M	NEG	UN	NEG	11.9						
**2077**	1/10/2022	1.5	M	NEG	UN	POS	34.31	75.59	64.01	54.68	56.65	84.94	49.34

ID	Capture Date	Age (years)	Sex	RT-PCR result	RT-PCR Ct	sVNT result	sVNT (inhibition %)	pVNT (NT_{50})					
								Wild type B.1	Alpha	Beta	Gamma	Delta	Omicron
**2078**	1/10/2022	0.5	M	NEG	UN	NEG	5.22						
**2079**	1/10/2022	2.5	M	NEG	UN	NEG	6.84						
**2080**	1/9/2022	0.5	M	NEG	UN	NEG	20.52						
**2081**	1/12/2022	2.5	M	NEG	UN	NEG	10.41						
**2082**	1/10/2022	1.5	M	NEG	UN	NEG	10.28						
**2083**	1/11/2022	0.5	M	NEG	UN	NEG	5.87						
**2084**	1/10/2022	0.5	M	NEG	UN	NEG	10.28						
**2085**	1/12/2022	3.5	M	NEG	UN	NEG	3.73						
**2086**	1/11/2022	1.5	M	NEG	UN	NEG	12.22						
**2087**	1/15/2022	0.5	M	NEG	UN	NEG	0.09						
**2088**	1/14/2022	2.5	M	NEG	UN	NEG	9.91						
**2089**	1/16/2022	1.5	M	POS	36.12	POS	78.74	1020	367.8	336.6	337.6	858.5	42.49
**2090**	1/15/2022	0.5	M	NEG	UN	NEG	8.98						
**2091**	1/11/2022	2.5	M	NEG	UN	NEG	0.94						
**2092**	1/25/2022	0.5	M	ND	ND	NEG	-5.83						
**2093**	1/16/2022	0.5	M	NEG	UN	NEG	6.19						
**2094**	1/14/2022	0.5	M	NEG	UN	ND	ND						
**2095**	1/20/2022	3.5	M	NEG	UN	NEG	3.14						
**2096**	1/16/2022	0.5	M	NEG	UN	NEG	6						
**2097**	1/15/2022	0.5	M	NEG	UN	NEG	12.16						
**2098**	1/17/2022	0.5	M	NEG	UN	NEG	5.2						
**2099**	1/12/2022	0.5	M	NEG	UN	NEG	13.52						
**2100**	1/17/2022	0.5	M	NEG	UN	NEG	9.87						
**2101**	1/23/2022	0.5	M	ND	ND	NEG	13.36						
**2102**	1/13/2022	0.5	M	NEG	UN	NEG	24.15						
**2103**	1/18/2022	0.5	M	POS	37	POS	93.99	725.8	910.5	797.5	875.7	1829	261.6
**2104**	1/24/2022	0.5	M	ND	ND	NEG	-3.05						
**2105**	1/18/2022	0.5	M	NEG	UN	NEG	-3.41						

ID	Capture Date	Age (years)	Sex	RT-PCR result	RT-PCR Ct	sVNT result	sVNT (inhibition %)	pVNT (NT_{50})					
								Wild type B.1	Alpha	Beta	Gamma	Delta	Omicron
**2106**	1/20/2022	2.5	M	NEG	UN	NEG	12.02						
**2107**	1/23/2022	0.5	M	ND	ND	NEG	3.05						
**2108**	1/27/2022	0.5	M	POS	22.5	NEG	5.11						
**2109**	1/21/2022	0.5	M	ND	ND	POS	83.14	351.3	291.2	75.84	113.4	501.9	43.48
**2110**	1/18/2022	0.5	M	NEG	UN	NEG	3.95						
**2111**	1/24/2022	0.5	M	ND	ND	NEG	6.64						
**2112**	1/25/2022	0.5	M	ND	ND	NEG	-10.49						
**2113**	1/24/2022	0.5	M	ND	ND	NEG	-8.52						
**2114**	1/25/2022	0.5	M	ND	ND	NEG	8.61						
**2115**	1/26/2022	0.5	M	ND	ND	NEG	4.3						
**2116**	1/25/2022	1.5	M	ND	ND	NEG	5.38						
**2117**	1/28/2022	0.5	M	NEG	UN	NEG	6.64						
**2118**	1/24/2022	0.5	M	ND	ND	NEG	2.42						
**2119**	1/26/2022	0.5	M	ND	ND	NEG	8.79						
**2120**	1/27/2022	0.5	M	NEG	UN	NEG	1.08						
**2121**	1/25/2022	0.5	M	ND	ND	NEG	6.19						
**2122**	1/26/2022	0.5	M	ND	ND	NEG	2.6						
**2123**	1/27/2022	2.5	M	NEG	UN	NEG	24.66						
**2124**	1/27/2022	1.5	M	NEG	UN	POS	92.38	1426	929.3	166.7	254.3	535.8	84.15
**2125**	1/30/2022	0.5	M	POS	31.1	NEG	4.13						
**2126**	1/30/2022	0.5	M	NEG	UN	NEG	1.79						
**40101**	1/19/2022	0.5	F	NEG	UN	NEG	-0.27						
**40105**	1/19/2022	0.5	F	NEG	UN	NEG	2.24						
**40109**	1/18/2022	0.5	F	ND	ND	NEG	-13.18						
**40115**	1/23/2022	0.5	F	ND	ND	NEG	11.75						
**40129**	1/20/2022	0.5	F	NEG	UN	NEG	8.34						

F: Female; M: Male; NEG: Negative; POS: Positive; ND: Not done; UN: Undetermined; sVNT: Virus neutralization using the surrogate virus neutralization assay; pVNT: Virus neutralization using pseudo virus.

Supplemental Table S2. Metadata associated with the phylogenetic tree presented in Figure 1B.

Identification number	Host	Collection date	Lineage	Location
NY WTD102[#]	White-Tailed Deer	1/6/2022	BA.1	New York, USA
NY WTD103[#]	White-Tailed Deer	1/6/2022	BA.1	New York, USA
NY WTD104[#]	White-Tailed Deer	1/6/2022	BA.1	New York, USA
NY WTD2067[#]	White-Tailed Deer	1/7/2022	BA.1	New York, USA
NY WTD2108[#]	White-Tailed Deer	1/27/2022	AY.39	New York, USA
NY WTD2103[#]	White-Tailed Deer	1/18/2022	AY.103	New York, USA
NY WTD2125[#]	White-Tailed Deer	1/30/2022	AY.39	New York, USA
NY WTD2089[#]	White-Tailed Deer	1/16/2022	AY.103	New York, USA
EPI_ISL_9347713	White-Tailed Deer	12/1/2021	AY.54	Arkansas, USA
EPI_ISL_9347714	White-Tailed Deer	12/1/2021	AY.45	Arkansas, USA
EPI_ISL_5804716	White-Tailed Deer	12/5/2020	B.1.119	Iowa, USA
EPI_ISL_5804724	White-Tailed Deer	12/8/2020	B.1.311	Iowa, USA
EPI_ISL_6425277	White-Tailed Deer	12/8/2020	B.1.119	Iowa, USA
EPI_ISL_5804743	White-Tailed Deer	12/8/2020	B.1.311	Iowa, USA
EPI_ISL_5804751	White-Tailed Deer	12/8/2020	B.1.311	Iowa, USA
EPI_ISL_6425281	White-Tailed Deer	12/8/2020	B.1.311	Iowa, USA
EPI_ISL_5804735	White-Tailed Deer	12/5/2020	B.1.264	Iowa, USA
EPI_ISL_5804765	White-Tailed Deer	1/9/2021	B.1.311	Iowa, USA
EPI_ISL_5804774	White-Tailed Deer	1/9/2021	B.1.311	Iowa, USA
EPI_ISL_6425287	White-Tailed Deer	1/9/2021	B.1.311	Iowa, USA
EPI_ISL_5804717	White-Tailed Deer	12/5/2020	B.1.311	Iowa, USA
EPI_ISL_6425280	White-Tailed Deer	12/5/2020	B.1.311	Iowa, USA
EPI_ISL_5804718	White-Tailed Deer	12/5/2020	B.1.311	Iowa, USA
EPI_ISL_5804719	White-Tailed Deer	12/5/2020	B.1.311	Iowa, USA
EPI_ISL_5804720	White-Tailed Deer	12/5/2020	B.1.311	Iowa, USA
EPI_ISL_5804721	White-Tailed Deer	12/5/2020	B.1.311	Iowa, USA
EPI_ISL_5804722	White-Tailed Deer	12/5/2020	B.1.311	Iowa, USA
EPI_ISL_6425271	White-Tailed Deer	12/5/2020	B.1.311	Iowa, USA
EPI_ISL_6425278	White-Tailed Deer	12/5/2020	B.1.311	Iowa, USA

Identification number	Host	Collection date	Lineage	Location
EPI_ISL_5804723	White-Tailed Deer	12/5/2020	B.1.311	Iowa, USA
EPI_ISL_5804725	White-Tailed Deer	1/9/2021	B.1.311	Iowa, USA
EPI_ISL_5804726	White-Tailed Deer	1/9/2021	B.1.311	Iowa, USA
EPI_ISL_6425273	White-Tailed Deer	12/6/2020	B.1	Iowa, USA
EPI_ISL_5804727	White-Tailed Deer	12/6/2020	B.1.240	Iowa, USA
EPI_ISL_5804728	White-Tailed Deer	12/6/2020	B.1.311	Iowa, USA
EPI_ISL_5804729	White-Tailed Deer	12/6/2020	B.1	Iowa, USA
EPI_ISL_6425274	White-Tailed Deer	12/6/2020	B.1.240	Iowa, USA
EPI_ISL_5804730	White-Tailed Deer	12/6/2020	B.1	Iowa, USA
EPI_ISL_5804731	White-Tailed Deer	12/10/2020	B.1.234	Iowa, USA
EPI_ISL_5804732	White-Tailed Deer	12/10/2020	B.1.234	Iowa, USA
EPI_ISL_5804733	White-Tailed Deer	12/10/2020	B.1.234	Iowa, USA
EPI_ISL_5804734	White-Tailed Deer	12/10/2020	B.1.234	Iowa, USA
EPI_ISL_5804736	White-Tailed Deer	12/8/2020	B.1	Iowa, USA
EPI_ISL_5804737	White-Tailed Deer	12/8/2020	B.1	Iowa, USA
EPI_ISL_5804738	White-Tailed Deer	12/8/2020	B.1	Iowa, USA
EPI_ISL_5804739	White-Tailed Deer	12/9/2020	B.1	Iowa, USA
EPI_ISL_5804740	White-Tailed Deer	12/9/2020	B.1	Iowa, USA
EPI_ISL_5804741	White-Tailed Deer	12/9/2020	B.1.400	Iowa, USA
EPI_ISL_5804742	White-Tailed Deer	12/9/2020	B.1.2	Iowa, USA
EPI_ISL_5804744	White-Tailed Deer	12/7/2020	B.1	Iowa, USA
EPI_ISL_5804745	White-Tailed Deer	12/7/2020	B.1.2	Iowa, USA
EPI_ISL_5804746	White-Tailed Deer	12/5/2020	B.1.2	Iowa, USA
EPI_ISL_5804747	White-Tailed Deer	12/5/2020	B.1.2	Iowa, USA
EPI_ISL_6425268	White-Tailed Deer	12/5/2020	B.1	Iowa, USA
EPI_ISL_5804748	White-Tailed Deer	12/9/2020	B.1	Iowa, USA
EPI_ISL_6425282	White-Tailed Deer	12/9/2020	B.1.2	Iowa, USA
EPI_ISL_5804749	White-Tailed Deer	12/7/2020	B.1.2	Iowa, USA
EPI_ISL_6425279	White-Tailed Deer	12/8/2020	B.1.2	Iowa, USA
EPI_ISL_6425275	White-Tailed Deer	12/7/2020	B.1.2	Iowa, USA
EPI_ISL_5804757	White-Tailed Deer	11/6/2020	B.1.2	Iowa, USA

Identification number	Host	Collection date	Lineage	Location
EPI_ISL_5804750	White-Tailed Deer	10/15/2020	B.1.2	Iowa, USA
EPI_ISL_5804752	White-Tailed Deer	12/22/2020	B.1.234	Iowa, USA
EPI_ISL_5804753	White-Tailed Deer	12/15/2020	B.1.234	Iowa, USA
EPI_ISL_5804754	White-Tailed Deer	12/28/2020	B.1.2	Iowa, USA
EPI_ISL_6425285	White-Tailed Deer	11/15/2020	B.1.2	Iowa, USA
EPI_ISL_6425269	White-Tailed Deer	11/30/2020	B.1.2	Iowa, USA
EPI_ISL_6425272	White-Tailed Deer	9/30/2020	B.1.2	Iowa, USA
EPI_ISL_5804755	White-Tailed Deer	11/29/2020	B.1.2	Iowa, USA
EPI_ISL_5804756	White-Tailed Deer	10/28/2020	B.1.2	Iowa, USA
EPI_ISL_5804758	White-Tailed Deer	10/31/2020	B.1.2	Iowa, USA
EPI_ISL_5804759	White-Tailed Deer	11/7/2020	B.1.2	Iowa, USA
EPI_ISL_5804760	White-Tailed Deer	11/8/2020	B.1.2	Iowa, USA
EPI_ISL_6425286	White-Tailed Deer	11/17/2020	B.1.2	Iowa, USA
EPI_ISL_5804761	White-Tailed Deer	11/24/2020	B.1.2	Iowa, USA
EPI_ISL_5804762	White-Tailed Deer	12/5/2020	B.1.2	Iowa, USA
EPI_ISL_5804763	White-Tailed Deer	12/10/2020	B.1.2	Iowa, USA
EPI_ISL_6425270	White-Tailed Deer	12/2/2020	B.1.2	Iowa, USA
EPI_ISL_6425284	White-Tailed Deer	9/28/2020	B.1.2	Iowa, USA
EPI_ISL_5804764	White-Tailed Deer	10/8/2020	B.1.2	Iowa, USA
EPI_ISL_5804766	White-Tailed Deer	11/24/2020	B.1.2	Iowa, USA
EPI_ISL_5804767	White-Tailed Deer	11/24/2020	B.1.2	Iowa, USA
EPI_ISL_5804768	White-Tailed Deer	11/24/2020	B.1.2	Iowa, USA
EPI_ISL_5804769	White-Tailed Deer	11/24/2020	B.1.2	Iowa, USA
EPI_ISL_5804770	White-Tailed Deer	11/24/2020	B.1.2	Iowa, USA
EPI_ISL_5804771	White-Tailed Deer	11/24/2020	B.1.2	Iowa, USA
EPI_ISL_6425283	White-Tailed Deer	11/24/2020	B.1.2	Iowa, USA
EPI_ISL_6425276	White-Tailed Deer	11/24/2020	B.1.2	Iowa, USA
EPI_ISL_5804772	White-Tailed Deer	11/24/2020	B.1.2	Iowa, USA
EPI_ISL_5804773	White-Tailed Deer	11/24/2020	B.1.2	Iowa, USA
EPI_ISL_5804775	White-Tailed Deer	11/24/2020	B.1.2	Iowa, USA
EPI_ISL_5804776	White-Tailed Deer	12/10/2020	B.1.2	Iowa, USA

Identification number	Host	Collection date	Lineage	Location
EPI_ISL_5804777	White-Tailed Deer	12/10/2020	B.1.2	Iowa, USA
EPI_ISL_5804778	White-Tailed Deer	12/10/2020	B.1.2	Iowa, USA
EPI_ISL_5804779	White-Tailed Deer	12/10/2020	B.1.2	Iowa, USA
EPI_ISL_5804780	White-Tailed Deer	12/10/2020	B.1.2	Iowa, USA
EPI_ISL_5804781	White-Tailed Deer	12/10/2020	B.1.2	Iowa, USA
EPI_ISL_5804782	White-Tailed Deer	12/10/2020	B.1.2	Iowa, USA
EPI_ISL_5804783	White-Tailed Deer	12/10/2020	B.1.2	Iowa, USA
EPI_ISL_5804784	White-Tailed Deer	12/10/2020	B.1.2	Iowa, USA
EPI_ISL_5804785	White-Tailed Deer	12/23/2020	B.1.2	Iowa, USA
EPI_ISL_5804786	White-Tailed Deer	11/17/2020	B.1.2	Iowa, USA
EPI_ISL_5804787	White-Tailed Deer	11/17/2020	B.1.2	Iowa, USA
EPI_ISL_6425266	White-Tailed Deer	11/18/2020	B.1.1	Iowa, USA
EPI_ISL_6425267	White-Tailed Deer	12/17/2020	B.1.2	Iowa, USA
EPI_ISL_9347708	White-Tailed Deer	11/20/2021	AY.75	Illinois, USA
EPI_ISL_9347715	White-Tailed Deer	11/21/2021	AY.3	Illinois, USA
EPI_ISL_9347716	White-Tailed Deer	12/6/2021	AY.3	Kansas, USA
EPI_ISL_9347717	White-Tailed Deer	12/3/2021	AY.3	Kansas, USA
EPI_ISL_9347727	White-Tailed Deer	12/1/2021	AY.119	Massachusetts, USA
EPI_ISL_9347728	White-Tailed Deer	11/30/2021	AY.119	Massachusetts, USA
EPI_ISL_9347725	White-Tailed Deer	12/10/2021	AY.25	Maine, USA
EPI_ISL_9347726	White-Tailed Deer	12/11/2021	AY.103	Maine, USA
EPI_ISL_9347711	White-Tailed Deer	12/7/2021	AY.103	Minnesota, USA
EPI_ISL_9347712	White-Tailed Deer	12/1/2021	AY.44	Minnesota, USA
EPI_ISL_9347723	White-Tailed Deer	11/20/2021	AY.103	North Carolina, USA
EPI_ISL_9347724	White-Tailed Deer	11/20/2021	AY.100	North Carolina, USA
EPI_ISL_9347709	White-Tailed Deer	12/2/2021	AY.25	New Jersey, USA
EPI_ISL_9347710	White-Tailed Deer	12/8/2021	AY.42	New Jersey, USA
EPI_ISL_9347708	White-Tailed Deer	11/24/2021	P.1	New York, USA
EPI_ISL_9347715	White-Tailed Deer	11/20/2021	AY.98.1	New York, USA
EPI_ISL_4878314	White-Tailed Deer	1/26/2021	B.1.582	Ohio, USA
EPI_ISL_4847029	White-Tailed Deer	1/28/2021	B.1.582	Ohio, USA

Identification number	Host	Collection date	Lineage	Location
EPI_ISL_4847030	White-Tailed Deer	1/28/2021	B.1.2	Ohio, USA
EPI_ISL_4878315	White-Tailed Deer	2/1/2021	B.1.2	Ohio, USA
EPI_ISL_4847031	White-Tailed Deer	2/1/2021	B.1.2	Ohio, USA
EPI_ISL_4878316	White-Tailed Deer	2/2/2021	B.1.2	Ohio, USA
EPI_ISL_4847032	White-Tailed Deer	2/16/2021	B.1.2	Ohio, USA
EPI_ISL_4847033	White-Tailed Deer	2/24/2021	B.1.596	Ohio, USA
EPI_ISL_4878317	White-Tailed Deer	2/25/2021	B.1.596	Ohio, USA
EPI_ISL_4878318	White-Tailed Deer	2/25/2021	B.1.596	Ohio, USA
EPI_ISL_4878319	White-Tailed Deer	2/25/2021	B.1.596	Ohio, USA
EPI_ISL_4878320	White-Tailed Deer	2/25/2021	B.1.596	Ohio, USA
EPI_ISL_4878321	White-Tailed Deer	2/25/2021	B.1.596	Ohio, USA
EPI_ISL_4878322	White-Tailed Deer	2/25/2021	B.1.596	Ohio, USA
EPI_ISL_9388142	White-Tailed Deer	11/20/2021	AY.25	Oklahoma, USA
EPI_ISL_9388143	White-Tailed Deer	11/20/2021	AY.25	Oklahoma, USA
EPI_ISL_9347718	White-Tailed Deer	11/28/2021	AY.107	Pennsylvania, USA
EPI_ISL_9347719	White-Tailed Deer	11/28/2021	B.1.1.7	Pennsylvania, USA
EPI_ISL_9388144	White-Tailed Deer	12/4/2021	AY.100	Tennessee, USA
EPI_ISL_9347720	White-Tailed Deer	12/4/2021	AY.47	Tennessee, USA
EPI_ISL_9347721	White-Tailed Deer	12/11/2021	AY.100	Virginia, USA
EPI_ISL_9347722	White-Tailed Deer	12/11/2021	AY.42	Virginia, USA
EPI_ISL_10168587	White-Tailed Deer	11/6/2021	AY.44	Quebec, Canada
EPI_ISL_10169675	White-Tailed Deer	11/7/2021	AY.44	Quebec, Canada
EPI_ISL_10170149	White-Tailed Deer	11/8/2021	AY.44	Quebec, Canada
EPI_ISL_10174207	White-Tailed Deer	12/1/2021	B.1	Ontario, Canada
EPI_ISL_10174960	White-Tailed Deer	12/4/2021	B.1	Ontario, Canada
EPI_ISL_10175390	White-Tailed Deer	11/3/2021	B.1	Ontario, Canada
EPI_ISL_10176284	White-Tailed Deer	12/1/2021	D.4	Ontario, Canada
EPI_ISL_9650712	White-Tailed Deer	10/14/2021	AY.103	Pennsylvania, USA
EPI_ISL_9650797	White-Tailed Deer	10/28/2021	AY.5	Pennsylvania, USA
EPI_ISL_9651200	White-Tailed Deer	10/22/2021	B.1.1.7	Pennsylvania, USA
EPI_ISL_9654019	White-Tailed Deer	11/27/2021	AY.88	Pennsylvania, USA

Identification number	Host	Collection date	Lineage	Location
EPI_ISL_9654444	White-Tailed Deer	11/27/2021	B.1.1.7	Pennsylvania, USA
EPI_ISL_9654523	White-Tailed Deer	11/28/2021	AY.88	Pennsylvania, USA
EPI_ISL_8769311	Environment - Composite wastewater sample	12/28/2021	BA.1.1	Vienna, Austria
EPI_ISL_9011265	Environment - Composite wastewater sample	1/2/2022	BA.1	Vienna, Austria
EPI_ISL_9011276	Environment - Composite wastewater sample	1/2/2022	BA.1	Vienna, Austria
EPI_ISL_9011282	Environment - Composite wastewater sample	1/2/2022	BA.1.1	Vienna, Austria
EPI_ISL_9011298	Environment - Composite wastewater sample	1/1/2022	BA.1.1	Vienna, Austria
EPI_ISL_9086969	Environment - Composite wastewater sample	1/9/2022	BA.1.1	Vienna, Austria
EPI_ISL_9049740	Hamster	1/8/2022	AY.4	Hong Kong
EPI_ISL_8542936	Hamster	11/1/2021	BA.1.1	Maharashtra, India
EPI_ISL_8372626	Human	12/22/2021	BA.1.1	New York, USA
EPI_ISL_8372629	Human	12/23/2021	BA.1.1	New York, USA
EPI_ISL_8372634	Human	12/26/2021	BA.1	New York, USA
EPI_ISL_8630855	Human	12/29/2021	BA.1	New York, USA
EPI_ISL_8630857	Human	12/29/2021	BA.1.1	New York, USA
EPI_ISL_8630859	Human	12/30/2021	BA.1	New York, USA
EPI_ISL_8630862	Human	12/30/2021	BA.1	New York, USA
EPI_ISL_8630863	Human	12/30/2021	BA.1	New York, USA
EPI_ISL_8630864	Human	12/31/2021	BA.1.1	New York, USA
EPI_ISL_8630865	Human	12/31/2021	BA.1	New York, USA
EPI_ISL_8630867	Human	12/31/2021	BA.1.1	New York, USA
EPI_ISL_8630868	Human	1/1/2022	BA.1.1	New York, USA
EPI_ISL_8630870	Human	1/1/2022	BA.1	New York, USA
EPI_ISL_8630871	Human	1/1/2022	BA.1	New York, USA
EPI_ISL_8630872	Human	1/1/2022	BA.1	New York, USA
EPI_ISL_8630873	Human	1/1/2022	BA.1	New York, USA
EPI_ISL_8630874	Human	1/1/2022	BA.1	New York, USA
EPI_ISL_8630875	Human	1/1/2022	BA.1	New York, USA
EPI_ISL_8630877	Human	1/2/2022	BA.1	New York, USA
EPI_ISL_8630891	Human	1/2/2022	BA.1	New York, USA

Identification number	Host	Collection date	Lineage	Location
EPI_ISL_8630892	Human	1/2/2022	BA.1	New York, USA
EPI_ISL_8630893	Human	1/2/2022	BA.1.1	New York, USA
EPI_ISL_8630894	Human	1/3/2022	BA.1.1	New York, USA
EPI_ISL_8630991	Human	1/3/2022	BA.1.1	New York, USA
EPI_ISL_8630997	Human	1/4/2022	BA.1	New York, USA
EPI_ISL_8630998	Human	1/4/2022	BA.1	New York, USA
EPI_ISL_8630999	Human	1/4/2022	BA.1	New York, USA
EPI_ISL_8631001	Human	1/4/2022	BA.1	New York, USA
EPI_ISL_8631002	Human	1/4/2022	BA.1	New York, USA
EPI_ISL_8631004	Human	1/5/2022	BA.1	New York, USA
EPI_ISL_8631029	Human	1/4/2022	BA.1	New York, USA
EPI_ISL_8631031	Human	1/5/2022	BA.1	New York, USA
EPI_ISL_8631032	Human	1/5/2022	BA.1	New York, USA
EPI_ISL_8631033	Human	1/5/2022	BA.1	New York, USA
EPI_ISL_8631034	Human	1/6/2022	BA.1	New York, USA
EPI_ISL_8631035	Human	1/3/2022	BA.1.1	New York, USA
EPI_ISL_8977456	Human	1/6/2022	BA.1.1	New York, USA
EPI_ISL_8977457	Human	1/6/2022	BA.1.1	New York, USA
EPI_ISL_8977536	Human	1/7/2022	BA.1	New York, USA
EPI_ISL_8977458	Human	1/7/2022	BA.1.1	New York, USA
EPI_ISL_8977459	Human	1/7/2022	BA.1	New York, USA
EPI_ISL_8977460	Human	1/8/2022	BA.1.1	New York, USA
EPI_ISL_8977525	Human	1/5/2022	BA.1	New York, USA
EPI_ISL_8977548	Human	1/8/2022	BA.1	New York, USA
EPI_ISL_8977461	Human	1/8/2022	BA.1	New York, USA
EPI_ISL_8977462	Human	1/9/2022	BA.1.1	New York, USA
EPI_ISL_8977541	Human	1/9/2022	BA.1	New York, USA
EPI_ISL_8977496	Human	1/10/2022	BA.1	New York, USA
EPI_ISL_8977497	Human	1/10/2022	BA.1.1	New York, USA
EPI_ISL_8977524	Human	1/10/2022	BA.1	New York, USA
EPI_ISL_8977498	Human	1/10/2022	BA.1.1	New York, USA

Identification number	Host	Collection date	Lineage	Location
EPI_ISL_8977499	Human	1/10/2022	BA.1	New York, USA
EPI_ISL_8977533	Human	1/10/2022	BA.1.1	New York, USA
EPI_ISL_8977500	Human	1/7/2022	BA.1	New York, USA
EPI_ISL_8977501	Human	1/8/2022	BA.1.1	New York, USA
EPI_ISL_8977502	Human	1/8/2022	BA.1.1	New York, USA
EPI_ISL_8977503	Human	1/9/2022	BA.1	New York, USA
EPI_ISL_8977504	Human	1/9/2022	BA.1	New York, USA
EPI_ISL_8977505	Human	1/9/2022	BA.1.1	New York, USA
EPI_ISL_8977542	Human	1/10/2022	BA.1.1	New York, USA
EPI_ISL_8977530	Human	1/10/2022	BA.1	New York, USA
EPI_ISL_8977534	Human	1/11/2022	BA.1	New York, USA
EPI_ISL_8977425	Human	1/11/2022	BA.1.1	New York, USA
ON-PHL-21-44225	Human	02/03/2022	D.4	Ontario, Canada

# Denotes the eight newly sequenced white-tailed deer SARS-CoV-2 genomes from this study. These new sequences are together with 64 representatively selected human-origin Omicron SARS-CoV-2 genomes circulating in New York City and Canada between December 2021 and February 2022 and 155 other white-tailed deer origin SARS-CoV-2 isolates, which are available in GISAID. An additional six environmentally derived (wastewater) origin and two hamster origin SARS-CoV-2 isolates were also included.
